# The Aquatic Ecosystem, a Good Environment for the Horizontal Transfer of Antimicrobial Resistance and Virulence-Associated Factors Among Extended Spectrum β-lactamases Producing *E. coli*

**DOI:** 10.3390/microorganisms8040568

**Published:** 2020-04-15

**Authors:** Lara Pérez-Etayo, David González, Ana Isabel Vitas

**Affiliations:** 1 Department of Microbiology and Parasitology, University of Navarra, 31008 Pamplona, Spain; dgonzalez@unav.es (D.G.); avitas@unav.es (A.I.V.); 2 Instituto de Investigación Sanitaria de Navarra (IDISNA), 31008 Pamplona, Spain

**Keywords:** virulence factor, antibiotic resistance, ESBL, wastewater, horizontal gene transfer

## Abstract

One of the main public health problems nowadays is the increase of antimicrobial resistance, both in the hospital environment and outside it (animal environment, food and aquatic ecosystems, among others). It is necessary to investigate the virulence-associated factors and the ability of horizontal gene transfer among bacteria for a better understanding of the pathogenicity and the mechanisms of dissemination of resistant bacteria. Therefore, the objective of this work was to detect several virulence factors genes (*fimA*, *papC*, *papG* III, *cnf1*, *hlyA* and *aer*) and to determine the conjugative capacity in a wide collection of extended-spectrum β-lactamases-producing *E. coli* isolated from different sources (human, food, farms, rivers, and wastewater treatment plants). Regarding virulence genes, *fimA*, *papC,* and *aer* were distributed throughout all the studied environments, *papG* III was mostly related to clinical strains and wastewater is a route of dissemination for *cnf1* and *hlyA*. Strains isolated from aquatic environments showed an average conjugation frequencies of 1.15 × 10^−1^ ± 5 × 10^−1^, being significantly higher than those observed in strains isolated from farms and food (*p* < 0.05), with frequencies of 1.53 × 10^−4^ ± 2.85 × 10^−4^ and 9.61 × 10^−4^ ± 1.96 × 10^−3^, respectively. The reported data suggest the importance that the aquatic environment (especially WWTPs) acquires for the exchange of genes and the dispersion of resistance. Therefore, specific surveillance programs of AMR indicators in wastewaters from animal or human origin are needed, in order to apply sanitation measures to reduce the burden of resistant bacteria arriving to risky environments as WWTPs.

## 1. Introduction

*E. coli* is one of the main causative agents of gastrointestinal and extra intestinal infections. This ubiquitous organism is a major element of the normal commensal microbiota in the human and animal intestinal tract and it has been found in soil, food, water, and vegetation [1]. The ability of *E. coli* to cause a variety of infectious diseases, such as sepsis, pneumonia, or urinary tract infections, is associated with the expression of specific virulence factors (VFs). In addition, the multidrug resistance profile of *E. coli* strains increases the risk of antimicrobial treatment failure in both humans and animals [2].

Resistance to antibiotics can occur by different processes, like the acquisitions of antimicrobial resistance genes (ARGs) via horizontal gene transfer (HGT). Several genetic mechanisms have been involved in the spread of ARGs, but conjugation is thought to have the greatest influence [3]. Furthermore, it has been reported that *E. coli* obtains antimicrobial resistance faster than other microorganisms [4]. It is especially relevant regarding the increase of β-lactam resistance in *E. coli* due to the production of extended spectrum β-lactamases (ESBL). Mobile Genetics Elements (MGSs) are able to spread ESBL associated genes by horizontal transfer to others Gram-negative bacteria [5].

Likewise, it is believed that the acquisition of several virulence factors via horizontal gene transfer provides an evolutionary pathway to pathogenicity [6]. VFs are important at the initial stages of infection (when the bacteria have to adapt to the host environment) and include adhesins (FimA, PapC, and PapG allele III), toxins (HlyA and Cnf1), and other proteins like siderophores (Aer) [7]. In the case of adherent structures, *fimA* encodes the major structural subunit of type 1 fimbriae and it is present in almost all *E. coli* strains and other members of the *Enterobacteriaceae* family [8]. However, *papC* and *papG* encode adhesin molecules that are found in P fimbriae and are especially linked to uropathogenic strains [9]. Regarding toxins, *cnf1* encodes for cytotoxic necrotizing factor type 1, a toxin secreted by some virulent strains associated with neonatal meningitis and urinary infections [10]. On the other hand, HlyA (cytotoxin hemolysin A or α hemolysin) is expressed with larger severity in infections caused by uropathogenic strains with a higher prevalence of kidney damage and bacteremia [11].

Previous studies performed by our research group [12,13,14,15,16] have provided us with a great collection of multidrug resistant ESBL-producing *E. coli* strains isolated from human, animal, food and water environments in Navarra (Spain). The objective of the present study was to (i) determine the virulence gene profiles and (ii) to determine the ability of horizontal gene transfer in a selection of ESBL-producing *E. coli* strains in order to a achieve a better understanding of the antibiotic resistance dissemination.

## 2. Materials and Methods

### 2.1. Strain Collection

From our own collection of ESBL-producing *Enterobacteriaceae* isolated in Navarra (Spain) from different sources, a total of 150 *E. coli* strains were selected for the study: human origin (including healthy volunteers (*n* = 13) and clinical cases (*n* = 36)), food products (*n* = 48), farms and feed (*n* = 20), and rivers and wastewater treatment plants [WWTPs] (*n* = 33). Having taken into account the available data from previous characterization [14,16,17], the following criteria were considered for the selection of strains: to show a multidrug resistant pattern (MDR), to carry different types of β-lactamase genes and belonging to different phylogenetic groups according to Clermont et al. [18] or different sequence types (including the ST131) following the scheme described by Wirth et al. [19]. Complete information of each strain (including the virulence genes and conjugation frequencies determined in this work) is presented in the Supplemental Material (Figures S1–S5).

### 2.2. Virulence Factor Gen Detection and Sequence Analysis

DNA extraction of the selected strains was performed with DNeasy^®^ Blood & Tissue kit (Qiagen, Barcelona, Spain), using a pre-treatment protocol for Gram-negative bacteria and following the manufacturer’s instruction. The quantity and quality of DNA was analyzed using a Nanodrop ND-1000 spectrophotometer (NanoDrop Technologies, Wilmington, DE, USA).

*E. coli* DNA extracts were tested by conventional PCR using the specific primers and conditions showed in Table 1, for the presence of VF genes that mediate adhesion (fimbrial adhesion genes, *fimA*, *papC,* and *papG* allele III), toxins (α-haemolysin *hlyA* and cytotoxic necrotizing factor *cnf1*) and siderophores (*aer*) [20,21].

Amplicons obtained were sequenced by the Macrogen EZ-Seq purification service (Macrogen Europe, Madrid, Spain) to confirm the presence of VF genes. Searches for DNA and protein homologies were carried out using the National Center for Biotechnology Information (http://www.ncbi.nlm.nih.gov/), using the BLAST program. Alignment of DNA and amino acids sequences was performed using Clustal Omega (http://www.ebi.ac.uk/Tools/msa/clustalo/).

### 2.3. Conjugation Assay

Conjugative transfer of ESBL genes among selected *E. coli* strains was studied using the mixed broth method [22]. *E. coli* DSM 9036 was used as a recipient strain. This strain is plasmid-free, streptomycin-resistant, and sensitive to β-lactams antibiotics (*F-, thr-1, ara-14, leuB6, Delta(gpt-proA)62, lacy,1 tsx-33, qsr-, supE44, galK2, lambda- rac-, hisG4(Oc), rfbD1, mgl-51, rpsL31, kdgK51, xyl-5, mtl-1, argE3(Oc), thi-1*). In the case of donor strains, as most of the *E. coli*-ESBL isolates were MDR, we selected as donor strains only those streptomycin sensitive (*n* = 70).

The donor and recipient strains were grown in BHI (Scharlab, Barcelona, Spain) at a concentration of approximately 1.0 × 10^9^ CFU/mL (overnight cultures, 37 °C). Equal volumes (5 mL) of cultures of the donor and the recipient strains were mixed (1:1) and incubated for 24 h at 37 °C. Transconjugants were selected on TSA agar (Scharlab) supplemented with streptomycin (100 µg/mL) and ampicillin (30 µg/mL) (Sigma-Aldrich, Madrid, Spain), to inhibit the growth of recipient and donors strains, respectively. After the incubation period (24 h at 37 °C), the number of colonies (CFU) was counted. Conjugation frequencies were expressed as the number of CFU of transconjugants relative to the number of CFU of donors. All experiments were performed in triplicate. The molecular characterization of the transconjugants was performed by PCR amplification of the genes involved in the resistances (*bla*CTX-M and *bla*TEM) [23,24].

### 2.4. Statistical Analysis

The results were subjected to statistical processing with the SPSS 15 software (SPSS Inc., Chicago, IL, USA), applying the Chi-square (X^2^) and ANOVA test with a level of significance of *p* < 0.05.

BioNumerics, version 7.6 (Applied Maths NV/bioMérieux, Sint-Martens-Latem, Belgium) was used to create the Multi-Dimensional Scaling (MDS) graphs. They were generated based on a distance matrix calculated by the Pearson correlation and unweighted pair-group method with arithmetic average (UPGMA) functions.

### 2.5. Informed Consent and Ethical Statement

Informed consent was obtained in all cases prior to collecting the samples (from parents in the case of participating children), using a template approved by the Ethical Committee Research of the University of Navarra (27 Jul 2018) [15].

## 3. Results

### 3.1. Prevalence of Virulence Factors Genes

Overall, all the isolates tested were positive for one or more VF genes (Figures S1–S5). In fact, the co-occurrence of several VFs in the same strain was frequent and most of the isolates contained two or three virulence genes (40.7% and 36%, respectively). Among the 150 tested strains, the genes encoding FimA (97.3%), Aer (72%) and PapC (60%) were the most commonly found and were detected in all studied environments (Table 2). Regarding *fimA*, the presence in farms and feed was significantly lower (*p* < 0.05) than that found in clinical cases and food products. Furthermore, the expression of *aer* in clinical samples was significantly higher than in rivers and WWTPs, food products, and healthy volunteers’ isolates. In contrast, the prevalence of *papC* was significantly different between all environments (*p* < 0.05) except for clinical, farms and feed and healthy volunteers’ samples.

**Figure 1:**
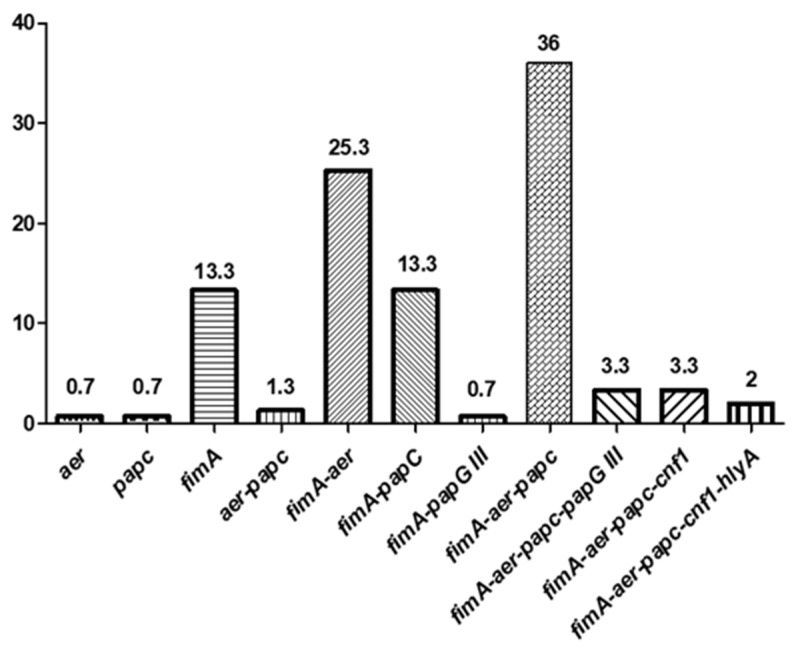
The co-occurrence (percentages) of several virulence factors (VFs) in ESBL-producing *E. coli*.

It is remarkable that the unique source in which all types of VF genes were detected were the clinical samples. In addition, those strains contain a greater number of virulence factors compared to those from the rest of the environments, but none of the isolates carried all the 6 studied genes. The three strains containing the *hlyA* gene (2%) were the only ones that presented 5 virulence factors (positive for *fimA*, *aer*, *papC*, *cnf1*, and *hlyA*). Likewise, the co-occurrence of *fimA-aer-papC* and *fimA-aer* was the most frequently reported (36% and 25.3%, respectively) (Figure 1).

### 3.2. Distribution of Virulence Genes among the Phylogenetic Groups

All the virulence genes were present among phylogroups B2 and B1 and most of them were detected in phylogenetic groups A and D (Table 3). It must be pointed out that gene toxins were found almost exclusively in A, B1, B2, and D groups. Adhesin PapC and toxin HlyA were more prevalent in group B2 as compared to the group D, in which the highest prevalence of toxin gene *cnf1* was found.

From the total of strains showing the ST131 (*n* = 16; all of them isolated from clinical cases and healthy volunteers), the vast majority (68.8%) were positive to *aer*, *papC*, and *fimA* (Figures S1 and S5). Furthermore, one clinical isolate ST131 was positive for 5 out of the 6 VF (*fimA*, *aer*, *papC*, *cnf1* and *hlyA*).

**Figure 2:**
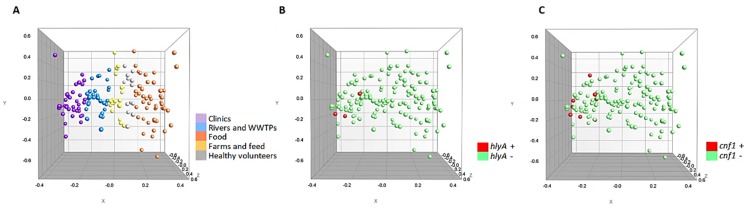
Multidimensional scaling graphs (MDS) for the 150 strains. (**A**) According to origin; (**B**) *hlyA* positive and negative strains (**C**) *cnf1* positive and negative strains.

Additionally, the multidimensional scaling graphs (MDS) showed in Figure 2 demonstrate that the 150 strains were homogeneously grouped according to the source of isolation (Figure 2A). Likewise, it can be seen that the *hlyA* and *cnf1* positive strains (Figure 2B,C) are strongly related, being very close in the variable space (X, Y and Z axis).

### 3.3. Horizontal Transfer of ESBL Genes

The 100% of the tested ESBL-producing *E. coli* strains (*n* = 70) were able to perform an efficient gene transfer, making the recipient strain (*E. coli* DSM 9036) resistant to ampicillin. Although the range of conjugation rates is nearly the same in samples from all origins, it was observed that strains isolated from aquatic environments showed significantly higher conjugation frequency values (*p* < 0.05), with an average value of 1.15 × 10^−1^ ± 5 × 10^−1^ (Table 4). In fact, the highest value was observed in a strain coming from a WWTP (2.35 ± 8.51 × 10^−2^), which confirms the potential risk of dissemination of ARG through these environments. On the other hand, the isolates from farms and feed and food products showed lower frequencies, with an average value of 1.53 × 10^−4^ ± 2.85 × 10^−4^ and 9.61 × 10^−4^ ± 1.96 × 10^−3^, respectively.

**Figure 3:**
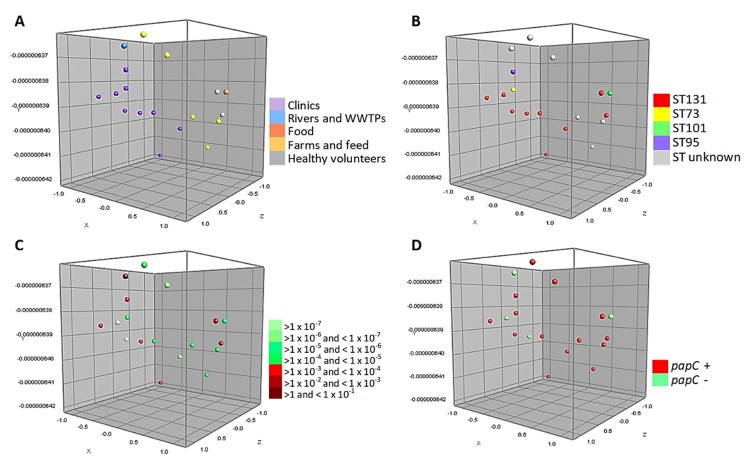
Multidimensional scaling graphs (MDS) for the 18 isolates B2, tested in the conjugation assays; (**A**) According to the distribution by origins; (**B**) MLST types; (**C**) Conjugation frequency ranges of these strains, and (**D**) Prevalence of *papC* genes.

Figure 3 shows the MDS of the 18 strains tested in the conjugation assays belonging to phylogroup B2 (associated with more virulent strains). These strains are closely associated, grouping homogeneously according to the source of isolation. Likewise, half of the isolates belong to ST131 and 39% of them are capable of performing a conjugation with a frequency range between >1 and < 1 × 10^−4^. In particular, it can be seen that 1 WWTPs isolate and 6 strains of human origin (clinical cases and healthy volunteers) have a high conjugation frequency. In addition, Figure 3D shows the relationship of the virulence factor *papC* with this phylogroup.

**Figure 4:**
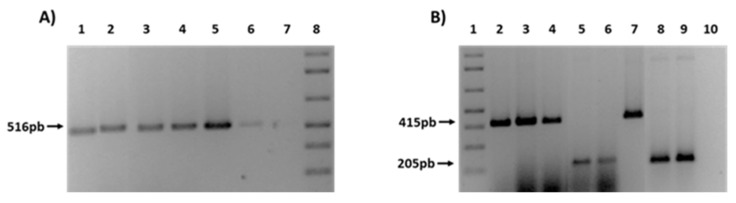
PCRs for the ESBL genes detection in transconjugants. (**A**) Presence of TEM-1 gene. 1: 1F; 2: 2F; 3: 3F; 4: 1R. 5-6: C + TEM-1; 7: C−; 8: 1Kb plus ladder. (**B**) Presence of CTX-M 1 and CTX-M9. 1: Kb plus ladder; 2: 1F; 3: 2F; 4: 2R; 5: 1W; 6: 2W. 7: C + CTX-M1; 8-9: C + CTX-M9; 10: C−.

Finally, in order to confirm the transfer of ESBL genes, different PCRs were performed for the detection of *bla*_CTX-M_ and *bla*_TEM_ genes in the transconjugants (Figure 4). As an example, Figure 4A shows the presence of TEM-1 gene in 4 transconjugants obtained from farm (F) and rivers (R) strain donors (1F, 2F, 3F, 1R). In a similar way, Figure 4B shows 5 transconjugants also from rivers (R) and WWTPs (W) (2R, 1W, 2W), that had acquired the type of CTX-M present in the donor strain (CTX-M1 or CTX-M9).

**Figure 5:**
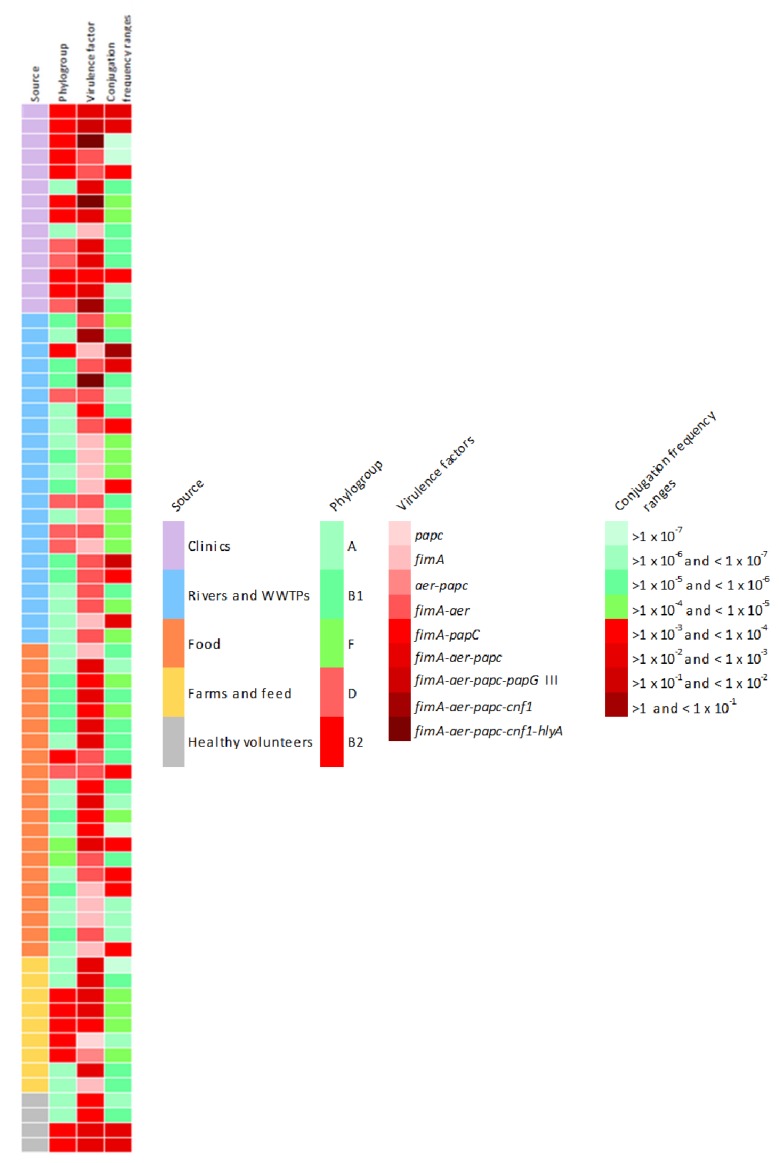
Phenotypic and genotypic characteristics of the 70 isolates tested in the conjugation assays. Red coloured boxes indicate the riskiest condition, according to the legend.

To sum up all the findings, Figure 5 shows the phenotypic and genotypic characteristics of the 70 isolates tested in the conjugation assays. Red colored boxes indicate the riskiest condition, according to the legend.

As we can see, although the highest conjugation frequency average have been observed in strains isolated from aquatic environments (1.15 × 10^−1^ ± 5 × 10^−1^), these strains only have one or two virulence factors, so clinical isolates contain the greatest amount of virulence factors studied and are related with the most pathogenic phylogroups. It must be taken into account that five strains isolated from clinical samples and healthy volunteers have been characterized as potentially pathogenic, since all of them belong to the ST131-B2 phylogroup, contain between 3 and 5 virulence factors and most of them have high conjugation rates.

## 4. Discussion

This study aimed to characterize the virulence and conjugative capacity of ESBL-producing *E. coli* isolates from human, food products, farm origin, and water environments, for a better understanding of the risk of dissemination of these resistant bacteria.

As expected, clinical isolates showed the highest prevalence of the studied virulence factor genes (encoding adhesins, siderophores, and toxins), in accordance with the main observed phylogroups in this origin (B1, B2 and D), which have been associated with more virulent strains (Figure 5). However, it must be noticed the presence of several VFs in strains isolated from aquatic environments, which could be related with the higher conjugation frequencies observed in those strains. Furthermore, it is remarkable that the majority of human strains showing the ST131 carried 3 or more VF genes, differing to Alonso et al. [25] who determined an apparent absence of classical virulence factors such as *papC*, *cnf1* and *hlyA*. In addition, the unique strain of the present study in which the co-occurrence of 5 VF genes was observed (*fimA*, *aer*, *papC*, *cnf1* and *hlyA*) was a clinical isolate ST131. Thus, the pathogenicity of ST131 *E. coli* isolates (causing infections in both community and hospital) has been associated to the large number of virulence-associated genes they contain [26].

Regarding adhesins, fimbriae have a fundamental role in the colonization (type I) and pathogenicity (type P) of extraintestinal infections caused by *E. coli* (such as urinary infections). Concerning *fimA*, the high prevalence observed (97.3%) suggests that type I fimbriae are widely distributed. Despite the fact that their presence is not limited to pathogenic strains [25], the expression of these fimbriae improves the virulence of uropathogenic *E. coli* [27]. Similarly, P fimbriae contribute to the virulence of uropathogenic strains by promoting bacterial colonization tissues and stimulating a host inflammatory response [28,29]. In the present study, we examined the genes associated with the outer membrane protein (*papC*) and *papG* III allele adhesin. The first one has been detected in all the studied environments, with a lower prevalence in aquatic environments (rivers and WWTPs, 15.2%). In contrast, *papG* III genes have been detected mainly in clinical strains, in accordance with the reported presence of this virulence factor in *E. coli* strains causing pyelonephritis or cystitis [30,31]. It has also been detected in two strains isolated from chicken and beef belonging to phylogroup D and carrying *intI*1 and *bla*_CTX-M14_. These results support the potential transmission of pathogenic *E. coli* through foods, having into account that an effective cell adhesion followed by invasion are the key events in pathogenicity [32]. In summary, type 1 fimbriae and P fimbriae can co-exist in the same microorganism, since the 6 positive strains for *papG* III also contain *fimA*. The reported association between *papG* III gene and genes encoding α-hemolysin (*hlyA*) and the cytotoxic necrotizing factor (*cnf1*) [28] has not been detected in this study.

With respect to siderophores, the *aer* gene has been detected in all environments but with higher prevalence in clinical isolates (91.6%). This gene encodes a bacterial iron chelating agent that allows *E. coli* to obtain iron from iron-poor environments such as the urinary tract [33]. The observed prevalence in average (72%) is similar to that reported by Raeispour and Ranjbar [34], but higher than that observed by Jalali et al. [35]. Some works indicate that there is a large variation in the *aer* frequencies, because the prevalence of this gene vary with phylogenetic groups, localization and clinical conditions [36]. Furthermore, Arisoy et al. [37] point out that *aer* might be one of the main contributors to the persistence of *E. coli* in the intestinal flora and Searle et al. [38] indicate that environmental *E. coli* isolates contain less genes associated with aerobactin. On the other hand, the clonal complexes ST155 linked with the phylogroup B1 is frequently associated with resistance and even with the ESBL phenotype in *E. coli* from human, animal and environmental sources. So, regarding our results and according with Alonso et al. [25], the virulence-associated factors genes of this ST was mainly *fimA* and *aer*.

Genes *hlyA* and *cnf1* encode toxins that can participate in the rupture of the epithelial barrier, allowing bacteria to pass from the digestive tract into the bloodstream and colonize different tissues, such as the urinary tract [39]. Hence, these factors are related to extraintestinal infections, mainly with uropathogenic strains and enterohemorrhagic *E. coli* that can cause diarrheal and haemolytic uremic syndrome [11]. In accordance with these observations, 5 clinical isolates coming from urine samples contain the *cnf1* gene. In addition, in 3 of them *hlyA* and *cnf1* genes coexist, coinciding with the observations of both genes in the same pathogenicity island [21]. In contrast to that published by Johnson et al. [40], the observed prevalence of *cnf1* in the present study is higher than *hlyA* (5.3% and 2%, respectively). It must be mentioned the prevalent presence of these virulence factors in clinical isolates, according with Cortés et al. [41]. However, these two factors have also been detected in 3 isolates from wastewater treatment plants (WWTPs). This would pose an especial risk, since these sources are considered to be the hotspots for the environmental dissemination of antibiotic resistant bacteria (ARB) [3]. Since cell adhesion, invasion, and the presence of toxins are the key events in pathogenicity, we can point out that the strains especially pathogenic are the 3 positives for *hlyA* gene, isolated from clinical samples and WWTP (Figure 5). However, it would be interesting to study whether these genetic profiles correspond to virulent phenotypes performing biofilm formation, adhesion and invasion assays.

Although WWTPs are designed to reduce the contamination and organic material of water, the presence of resistant bacteria in the effluents has been reported [13,42]. In fact, the environmental conditions in these plants favor the proliferation of ARB, the dispersion of ARG and the production of strong biofilm that increases the capacity to colonize the sewer system [43,44]. High conjugation rates have been reported in bacterial biofilms [45], which together with the observed conjugation frequencies in aquatic strains, poses WWTPs as risky environments for the transmission of AMR. As indicated by WHO [46], the appropriate management and treatment of sewage is an essential action for the prevention of the spread of different human diseases. Therefore, the first step to combat environmental dissemination routes of AMR is to ensure that at least basic sanitation needs are met.

However, scientific knowledge has not yet progressed to establish the objectives for estimating the risks of ARB and ARG abundance in wastewater. Assessing the different risks to which human populations may be exposed and determining pollutants concentrations should be one of the main objectives [47]. Nonetheless, it is difficult to imagine a risk assessment framework that includes all complex gene transfer events, which may take place from environmental bacteria to human or animal pathogens [48].

Resistance monitoring data in humans and farm animals in several regions should be used to provide necessary information and to select the specific gen markers under study. For that, it would be valuable to determine whether simple indicators represent a broadly risk. According to Gillings et al. [49] the integrase class 1 *intI*1 could be used as a promising indicator for anthropogenic ARG contamination, because they have a high clinical relevance. In fact, the 92% of the strains tested in this study carried the aforementioned gene *intI*1 (Figures S1–S5). Another thing that would be valuable to measure is the rates at which the horizontal gene transfer occurs in the WWTPs. However, this is still an important knowledge gap [47]. In this sense, it has been reported that conjugation is an extremely effective mechanism for dissemination of ESBLs [50]. Our data reinforce the hypothesis of ARG dissemination in aquatic environments through this mechanism, because higher conjugation frequencies have been observed in strains isolated from these environments, especially from WWTPs. In addition, conjugation experiments showed that these genes are probably located in the same transferable plasmid. In fact, the 4 strains with higher conjugation frequencies, contain the integrase class 1 *intI*1 gene and different insertion sequences (*ISEcp1*, IS26, IS903) (Figures S1–S5). Nevertheless, very little is known about the health risks posed by exposure to commensal or environmental bacteria that carry mobile ARGs [47]. The conjugation process may occur in many types of ecosystems, but in food environments can have very serious consequences, due to the mobilization of virulence genes and toxins [51]. As good news, our results indicate that the average conjugation frequencies in bacteria isolated from food products was not very high (9.61 × 10^−4^ ± 1.96 × 10^−3^). The food chain is one of the main routes for the introduction of resistant bacteria into the gastrointestinal tract, where genes can be transferred between pathogenic and opportunistic bacteria (as shown from our data from healthy volunteers).

In conclusion, this study has provided information on genotypes related to resistance, virulence and conjugation capacity of ESBL-producing *E. coli* isolated from different environments. The obtained results point out the important role of the aquatic environment for virulence gene exchange and resistance dissemination. Therefore, it would be necessary to control the presence of multidrug resistant bacteria (o superbug) in risky environments such as wastewater treatment plants (WWTPs) to ensure the effectiveness of antibiotics for public health.

## Figures and Tables

**Table 1:** Primers and conditions used for the amplification of virulence factors genes.

Target	Primer	Sequence (5′-3′)	Size (bp)	T (°C)	Reference
*fimA*	fimA-Fw	GTTGTTCTGTCGGCTCTGTC	447	55	[21]
	fimA-Rv	ATGGTGTTGGTTCCGTTATTC			
*papG*III	papG-Fw	CATTTATCGTCCTCAACTTAG	482	55	[21]
	papG-Rv	AAGAAGGGATTTTGTAGCGTC			
*papC*	papC-Rw	GACGGCTGTACTGCAGGGTGTGGCG	382	63	[20]
	papC-Rv	ATATCCTTTCTGCAGGGATGCAATA			
*aer*	aer-Fw	TACCGGATTGTCATATGCAGACCGT	602	63	[20]
	aer-Rv	AATATCTTCCTCCAGTCCGGAGAAG			
*hlyA*	hlyA-Fw	AACAAGGATAAGCACTGTTCTGGCT	1117	63	[20]
	hlyA-Rv	ACCATATAAGCGGTCATTCCCGTCA			
*cnfI*	cnfI-Fw	AAGATGGAGTTTCCTATGCAGGAG	498	63	[20]
	cnfI-Rv	CATTCAGAGTCCTGCCCTCATTATT			

**Table 2:** Prevalence of virulence-associated genes among extended spectrum β-lactamase (ESBL)-producing *E. coli* from different sources.

Gene	Number of Isolates (%)					
	Clinical Cases	Healthy Volunteers	Food Products	Farms and Feed	Rivers and WWTPs	Total
*fimA*	36 (100) ^a^	13 (100)	48 (100) ^b^	17 (85) ^a,b^	32 (97)	146 (97.3)
*papG III*	4 (11.1)	0	2 (4.1)	0	0	6 (4)
*papC*	30 (83.3) ^c,d^	13 (100) ^g,i^	24 (50) ^d,e,h,i^	18 (90) ^f,h^	5 (15.2) ^c,e,f,g^	90 (60)
*aer*	33 (91.6) ^j,k,l^	9 (69.2) ^l^	28 (58.3) ^k^	15 (75)	23 (69.7) ^j^	108 (72)
*hlyA*	2 (5.5)	0	0	0	1 (3)	3 (2)
*cnf1*	5 (13.8)	0	0	0	3 (9)	8 (5.3)

a–l: Same letters represents statistically significant values (*p* < 0.05) between these groups.

**Table 3:** Distribution of virulence genes among phylogenetic groups of ESBL-producing *E. coli.*

VF		Number of Isolates (% of Total)							
	Gene	A	B1	B2	D	C	F	Clade I	Unknown
		(*n* = 44)	(*n* = 27)	(*n* = 30)	(*n* = 36)	(*n* = 3)	(*n* = 6)	(*n* = 1)	(*n* = 3)
Adhesins	*fimA*	43 (97.7)	27 (100)	28 (93.3)	35 (97.2)	3 (100)	6 (100)	1 (100)	3 (100)
	*papC*	24 (54.4)	16 (59.3)	25 (83.3)	21 (58.3)	2 (66.6)	1 (16.6)	-	1 (33.3)
	*papG III*	2 (4.5)	1 (3.7)	1 (3.3)	2 (5.5)	-	-	-	-
Siderophore	*aer*	25 (56.8)	17 (63)	24 (80)	32 (88.8)	3 (100)	6 (100)	-	1 (33.3)
Toxins	*hlyA*	-	1 (3.7)	2 (6.6)	-	-	-	-	-
	*cnf1*	1 (2.27)	1 (3.7)	2 (6.6)	4 (11.1)	-	-	-	-

**Table 4:** Conjugation frequencies of ESBL-producing *E. coli* according to their origin.

Origin	Conjugation Frequency Average ± Sd	Conjugation Frequency Range
Rivers and WWTPs	1.15 × 10^−1^ ± 5 × 10^−1^	2.35–3.37 × 10^−6^
Healthy volunteers	3.38 × 10^−2^ ± 4.20 × 10^−2^	4.81× 10^−2^–2.28 × 10^−6^
Clinical cases	2.64 × 10^−3^ ± 5.82 × 10^−3^	1.19 × 10^−2^–9.08 × 10^−7^
Farms and feeds	1.53 × 10^−4^ ± 2.85 × 10^−4^	1.03 × 10^−4^–9.14 × 10^−7^
Food products	9.61 × 10^−4^ ± 1.96 × 10^−3^	1.16 × 10^−3^–3.59 × 10^−7^

## Supplementary Materials

The following are available online at https://www.mdpi.com/2076-2607/8/4/568/s1. Figure S1. Phenotypic and genotypic characteristics of isolates from clinical cases (*n* = 36), Figure S2. Phenotypic and genotypic characteristics of isolates from healthy volunteers (*n* = 13), Figure S3. Phenotypic and genotypic characteristics of isolates from aquatic environments (*n* = 33), Figure S4. Phenotypic and genotypic characteristics of isolates from food (*n* = 48), Figure S5. Phenotypic and genotypic characteristics of isolates of farm origin (*n* = 20).
