# Supplementary material for: The Aquatic Ecosystem, a Good Environment for the Horizontal Transfer of Antimicrobial Resistance and Virulence-Associated Factors Among Extended Spectrum β-lactamases Producing E. coli
Source: Microorganisms. 2020 Apr 15;8(4):568. doi: 10.3390/microorganisms8040568 (PMC7232254; doi:10.3390/microorganisms8040568)
Supplement: Supplementary file 1 [file microorganisms-08-00568-s001.pdf]

**Figure S1**. Phenotypic and genotypic characteristics of isolates from clinical cases (*n*=36)

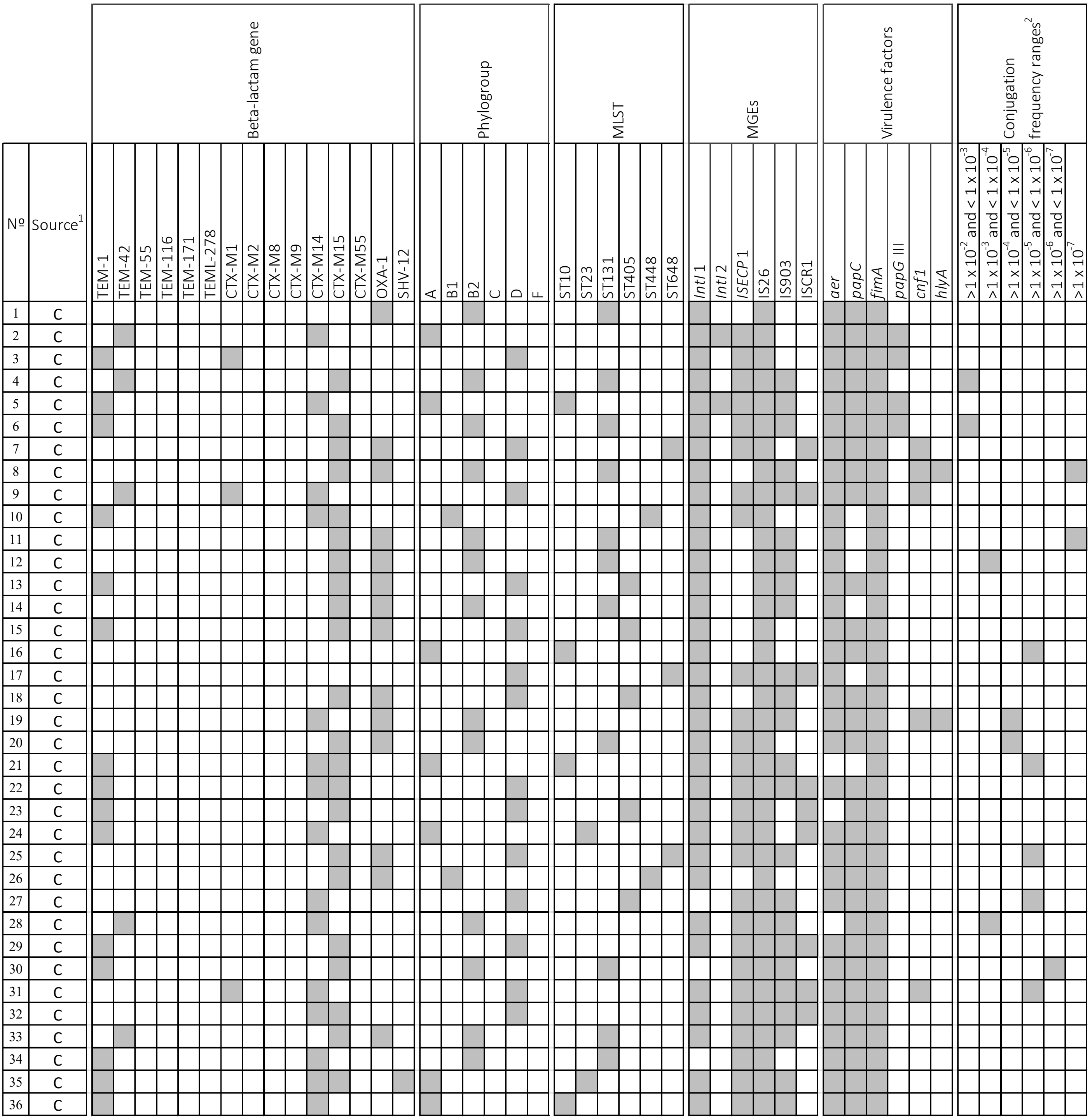


[1] C: Clinic case

[2] Conjugation frequencies: Average of three independent experiments

**Figure S2**. Phenotypic and genotypic characteristics of isolates from healthy volunteers (*n*=13)

| Nº | Source[1] | Sex[2]/age range | Beta-lactam gene | | | | | | | | | | | | | | Phylogroup | | | | | | MLST | | | | MGEs | | | | | | Virulence factors | | | | | | Conjugation frequency ranges[3] | | |
|---|---|---|---|---|---|---|---|---|---|---|---|---|---|---|---|---|---|---|---|---|---|---|---|---|---|---|---|---|---|---|---|---|---|---|---|---|---|---|---|---|---|
| | | | TEM-1 | TEM-42 | TEM-55 | TEM-116 | TEM-171 | TEML-278 | CTX-M1 | CTX-M2 | CTX-M8 | CTX.M9 | CTX-M14 | CTX-M15 | OXA-1 | SHV-12 | A | B1 | B2 | C | D | F | ST38 | ST131 | ST971 | ST2404 | *Int* I1 | *IntI* 2 | *ISECP* 1 | IS26 | IS903 | ISCR1 | *aer* | *papC* | *fimA* | *papG* III | *cnf1* | *hlyA* | $>1 x 10^{-2}$ and $< 1 x 10^{-3}$ | $>1 x 10^{-5}$ and $< 1 x 10^{-6}$ | $>1 x 10^{-6}$ and $< 1 x 10^{-7}$ |
| 1 | HV | $M_{40-65}$ | | | | ■ | | | | | | | ■ | | | | | ■ | | | | | | | ■ | | ■ | | | ■ | ■ | | ■ | ■ | ■ | | | | | | |
| 2 | HV | $F_{5-18}$ | | | | ■ | | | | | | | ■ | | | | ■ | | | | | | | | | ■ | ■ | | | ■ | ■ | ■ | ■ | ■ | ■ | | | | | | |
| 3 | HV | $F_{18-40}$ | | | | | ■ | | ■ | | | | | | | | | | | | ■ | | ■ | | | | ■ | | | ■ | ■ | | | ■ | ■ | | | | | | |
| 4 | HV | $F_{18-40}$ | | | | | ■ | | ■ | | | | | | | | | | | | | | | | | | ■ | ■ | | ■ | ■ | | | ■ | ■ | | | | | | |
| 5 | HV | $M_{18-40}$ | | | | | ■ | | | | | | ■ | ■ | | | | | ■ | | | | | ■ | | | ■ | | ■ | ■ | | | ■ | ■ | ■ | | | | | | |
| 6 | HV | $F_{18-40}$ | | | | | | | | | | | ■ | | | | ■ | | | | | | | | | | ■ | | ■ | ■ | | | | ■ | ■ | | | | | | ■ |
| 7 | HV | $F_{18-40}$ | | | | | ■ | | | | | | | | | | ■ | | | | | | | | | | ■ | | ■ | ■ | | | | ■ | ■ | | | | | ■ | |
| 8 | HV | $M_{40-65}$ | | | | | ■ | | | | | | | ■ | | | | | ■ | | | | | ■ | | | ■ | | ■ | ■ | | | ■ | ■ | ■ | | | | ■ | | |
| 9 | HV | $M_{40-65}$ | | | | | ■ | | | | | | | ■ | | | | | ■ | | | | | ■ | | | ■ | | ■ | ■ | ■ | | ■ | ■ | ■ | | | | ■ | | |
| 10 | HV | $M_{>65}$ | | | | | | | | | | | ■ | | | | | | ■ | | | | | ■ | | | ■ | | | ■ | ■ | | ■ | ■ | ■ | | | | | | |
| 11 | HV | $M_{5-18}$ | | | | | | | | | | | ■ | | | | | | ■ | | | | | ■ | | | ■ | | | ■ | ■ | | ■ | ■ | ■ | | | | | | |
| 12 | HV | $F_{5-18}$ | | | | | | | | | | | | ■ | | | | | | ■ | | | | | | | ■ | | | ■ | | | ■ | ■ | ■ | | | | | | |
| 13 | HV | $F_{>65}$ | | | | | | | | | | | | | | | | | | | ■ | | | | | | ■ | | | ■ | | | ■ | ■ | ■ | | | | | | |

[1] HV: healthy volunteers

[2] F: Female; M: Male

[3] Conjugation frequencies: Average of three independent experiments

**Figure S3.** Phenotypic and genotypic characteristics of isolates from aquatic environments (*n*=33)

| Nº | Source[1] | Beta-lactam gene | | | | | | | | | | | | | | |
|---|---|---|---|---|---|---|---|---|---|---|---|---|---|---|---|---|
| | | TEM-1 | TEM-42 | TEM-55 | TEM-116 | TEM-171 | TEML-278 | CTX-M1 | CTX-M2 | CTX-M8 | CTX-M9 | CTX-M14 | CTX-M15 | CTX-M55 | OXA-1 | SHV-12 |
| 1 | WWTP | | | | | | | | | | | ■ | | | | |
| 2 | WWTP | | | | | | | | | | | | ■ | | ■ | |
| 3 | WWTP | | | | | | | | | | | | ■ | | | |
| 4 | WWTP | | | | | | | ■ | | | | | | | | |
| 5 | WWTP | ■ | | | | | | | | | | | ■ | | ■ | |
| 6 | WWTP | ■ | | | | | | | | | | ■ | | | | |
| 7 | WWTP | | | | | | | | | | | | ■ | | | |
| 8 | WWTP | | | | | | | | | | | ■ | | | | |
| 9 | WWTP | | | | | | | ■ | | | | | | | | |
| 10 | WWTP | | | | | | | ■ | | | | | | | | |
| 11 | WWTP | ■ | | | | | | | | | | ■ | | | | |
| 12 | WWTP | | | | | | | ■ | | | | | | | | |
| 13 | WWTP | | | | | | | | | | | | ■ | | | |
| 14 | WWTP | | ■ | | | | | | | | | | | | | ■ |
| 15 | WWTP | | | | | | | | | | | | ■ | | | |
| 16 | WWTP | ■ | | | | | | | | | | ■ | | | | |
| 17 | WWTP | | | | | | | | | | | ■ | | | | |
| 18 | WWTP | | | | | | | | | | | | ■ | | ■ | |
| 19 | WWTP | | | | | | | ■ | | | | | | ■ | | |
| 20 | WWTP | | | | | | | | | | | | | | | |
| 21 | R | ■ | | | | | | ■ | | | | | | | | |
| 22 | R | | | | | | | | | | | ■ | | | | |
| 23 | R | | | | | | | | | | | | ■ | | | |
| 24 | R | ■ | | | | | | ■ | | | | | | | | |
| 25 | R | | | | | | | ■ | | | | | | | | |
| 26 | R | | ■ | | | | | ■ | | | | | | | | |
| 27 | R | ■ | | | | | | | | | | | ■ | | | |
| 28 | R | ■ | | | | | | | | | | | ■ | | | |
| 29 | R | | | | | | | | | | | | | | | ■ |
| 30 | R | ■ | | | | | | | | | | | ■ | | | |
| 31 | R | | | | | | | | | | | ■ | | | | |
| 32 | R | | | | | | | | | | | ■ | | | | |
| 33 | R | | | | | | | | | | | ■ | | | | |

| Nº | Phylogroup | | | | | | MLST | | | | | | |
|---|---|---|---|---|---|---|---|---|---|---|---|---|---|
| | A | B1 | B2 | C | D | F | ST10 | ST38 | ST46 | ST155 | ST168 | ST350 | ST469 |
| 1 | | ■ | | | | | | | | ■ | | | |
| 2 | ■ | | | | | | ■ | | | | | | |
| 3 | | | ■ | | | | | | | | | | |
| 4 | | ■ | | | | | | | | | | | |
| 5 | | ■ | | | | | | | | | | | |
| 6 | | | | | ■ | | | | | | | ■ | |
| 7 | | | | | ■ | | | | | | | | |
| 8 | ■ | | | | | | | | ■ | | | | |
| 9 | ■ | | | | | | | | | | ■ | | |
| 10 | | | | | ■ | | | | | | | | |
| 11 | ■ | | | | | | ■ | | | | | | |
| 12 | | ■ | | | | | | | | | | | ■ |
| 13 | ■ | | | | | | | | ■ | | | | |
| 14 | ■ | | | | | | | | | | | | |
| 15 | | ■ | | | | | | | | | | | |
| 16 | | | | | ■ | | | | | | | | |
| 17 | | | | | ■ | | | | | | | | |
| 18 | | | | | ■ | | | | | | | | |
| 19 | | | | | ■ | | | | | | | | ■ |
| 20 | ■ | | | | | | | | | | | | |
| 21 | | | | | ■ | | | | | | | | |
| 22 | | | | | ■ | | | | | | | | |
| 23 | | | | | ■ | | | | | | | | |
| 24 | | ■ | | | | | | | | | | | |
| 25 | | | | | ■ | | | ■ | | | | | |
| 26 | ■ | | | | | | ■ | | | | | | |
| 27 | | ■ | | | | | | | | | | | |
| 28 | ■ | | | | | | | | | | | | |
| 29 | | ■ | | | | | | | | | | | |
| 30 | ■ | | | | | | | | | | | | |
| 31 | | | | | ■ | | | | | | | | |
| 32 | ■ | | | | | | | | ■ | | | | |
| 33 | ■ | | | | | | | | ■ | | | | |

| Nº | MGEs | | | | | | Virulence factors | | | | | | Conjugation frequency ranges[2] | | | | | | |
|---|---|---|---|---|---|---|---|---|---|---|---|---|---|---|---|---|---|---|---|
| | *IntI* 1 | *IntI* 2 | *ISECP* 1 | IS26 | IS903 | ISCR1 | *aer* | *papC* | *fimA* | *papG* III | *cnf1* | *hlyA* | >1 and < $1 \times 10^{-1}$ | > $1 \times 10^{-1}$ and < $1 \times 10^{-2}$ | > $1 \times 10^{-2}$ and < $1 \times 10^{-3}$ | > $1 \times 10^{-3}$ and < $1 \times 10^{-4}$ | > $1 \times 10^{-4}$ and < $1 \times 10^{-5}$ | > $1 \times 10^{-5}$ and < $1 \times 10^{-6}$ | > $1 \times 10^{-6}$ and < $1 \times 10^{-7}$ |
| 1 | ■ | | ■ | ■ | | | ■ | | ■ | | | | | | | | ■ | | |
| 2 | ■ | | ■ | ■ | ■ | | ■ | ■ | ■ | | ■ | | | | | | | ■ | |
| 3 | ■ | | ■ | ■ | ■ | | | | ■ | | | | ■ | | | | | | |
| 4 | ■ | | ■ | ■ | ■ | | ■ | | ■ | | | | | | ■ | | | | |
| 5 | ■ | | ■ | ■ | ■ | | ■ | ■ | ■ | | ■ | ■ | | | | | | ■ | |
| 6 | ■ | | ■ | ■ | | | ■ | ■ | ■ | | ■ | | | | | | | | |
| 7 | ■ | | ■ | ■ | | | ■ | | ■ | | | | | | | | | | ■ |
| 8 | ■ | | ■ | ■ | ■ | | | ■ | ■ | | | | | | | | | ■ | |
| 9 | ■ | | ■ | ■ | ■ | | ■ | | ■ | | | | | | | ■ | | | |
| 10 | ■ | | ■ | ■ | | | ■ | | ■ | | | | | | | | | | |
| 11 | ■ | | ■ | ■ | | | | | ■ | | | | | | | ■ | | | |
| 12 | ■ | | ■ | ■ | ■ | | | | ■ | | | | | | | ■ | | | |
| 13 | | | ■ | ■ | ■ | | | | ■ | | | | | | | ■ | | | |
| 14 | ■ | | ■ | ■ | ■ | | ■ | | ■ | | | | | | | | | | |
| 15 | ■ | | ■ | ■ | ■ | | | | ■ | | | | | | ■ | | | | |
| 16 | ■ | | ■ | ■ | ■ | | ■ | | ■ | | | | | | | | | | |
| 17 | ■ | | ■ | ■ | ■ | | ■ | | ■ | | | | | | | | | | |
| 18 | | | ■ | | | | ■ | | | | | | | | | | | | |
| 19 | ■ | | ■ | ■ | ■ | | ■ | | ■ | | | | | | | | | ■ | |
| 20 | ■ | | ■ | ■ | ■ | | | | ■ | | | | | | | | ■ | | |
| 21 | ■ | | ■ | ■ | | | ■ | | ■ | | | | | | | | ■ | | |
| 22 | ■ | | ■ | ■ | ■ | | ■ | | ■ | | | | | | | | | | |
| 23 | ■ | | ■ | ■ | | | | ■ | ■ | | | | | | | | ■ | | |
| 24 | ■ | | ■ | ■ | ■ | | ■ | | ■ | | | | | ■ | | | | | |
| 25 | ■ | | ■ | ■ | ■ | | ■ | | ■ | | | | | | | | | | |
| 26 | ■ | | ■ | ■ | ■ | | ■ | | ■ | | | | | | | | | | |
| 27 | ■ | | | ■ | ■ | | ■ | | ■ | | | | | | | ■ | | | |
| 28 | ■ | | ■ | ■ | | | ■ | | ■ | | | | | | | | | ■ | |
| 29 | | ■ | | ■ | ■ | | | | ■ | | | | | | | | | | |
| 30 | ■ | | ■ | ■ | ■ | ■ | ■ | | ■ | | | | | | | | ■ | | |
| 31 | ■ | | ■ | ■ | ■ | | ■ | | ■ | | | | | | | | | | |
| 32 | ■ | | ■ | ■ | ■ | | | | ■ | | | | | | ■ | | | | |
| 33 | ■ | | ■ | ■ | | | ■ | | ■ | | | | | | | | ■ | | |

[1] WWTP: wastewater treatment plant; R: river.

[2] Conjugation frequencies: Average of three independent experiments

**Figure S4.** Phenotypic and genotypic characteristics of isolates from food ($n$=48)

| | | Beta-lactam gene | | | | | | | | | | | | | | | Phylogroup | | | | | | MLST | | | | | | | | | MGEs | | | | | | Virulence factors | | | | | | Conjugation frequency ranges[2] | | | | |
|---|---|---|---|---|---|---|---|---|---|---|---|---|---|---|---|---|---|---|---|---|---|---|---|---|---|---|---|---|---|---|---|---|---|---|---|---|---|---|---|---|---|---|---|---|---|---|---|---|
| Nº | Source[1] | TEM-1 | TEM-42 | TEM-55 | TEM-116 | TEM-171 | TEML-278 | CTX-M1 | CTX-M2 | CTX-M8 | CTX-M9 | CTX-M14 | CTX-M15 | CTX-M55 | OXA-1 | SHV-12 | A | B1 | B2 | C | D | F | ST10 | ST23 | ST46 | ST101 | ST117 | ST155 | ST168 | ST354 | ST648 | Int1 | Intl2 | *ISECP* 1 | IS26 | IS903 | ISCR1 | aer | papC | fimA | papG III | cnf1 | hlyA | $>1 \times 10^{-3}$ and $< 1 \times 10^{-4}$ | $>1 \times 10^{-4}$ and $< 1 \times 10^{-5}$ | $>1 \times 10^{-5}$ and $< 1 \times 10^{-6}$ | $>1 \times 10^{-6}$ and $< 1 \times 10^{-7}$ | $>1 \times 10^{-7}$ |
| 1 | C | | | | | | | | | | |■| | | | | | | | |■| | | | | | | | | |■|■|■|■|■|■| |■| |■| | | | | | | | |
| 2 | P | | | | | | | | | | |■| | | | |■| | | | | | | |■| | | | | | | | | |■|■| | | |■| | | | | |■| | |
| 3 | C |■| | | | | | | | | |■| | | | |■| | | | | | | |■| | | | | | |■| |■|■|■| |■|■|■| | | | | | |■| |
| 4 | C | | | | | | |■| | | | | | | | | |■| | | | | | | | | |■| | | |■| |■|■|■| | |■|■| | | | |■| | | |
| 5 | C | | | | | | |■| | | | | | | | | | |■| | | | | | |■| | | | | |■| |■|■|■| |■|■|■| | | | | |■| | |
| 6 | P | | | | | | |■| | | | | | | | | |■| | | | | | | | | |■| | | |■| |■|■| | |■|■|■| | | | |■| | | |
| 7 | P | |■| | | | | | | | | | | | | | |■| | | | | | | | | | | | | | | | |■|■| | |■|■| | | | | | | | |
| 8 | C |■| | | | | | | | | | | | | |■| | | | |■| | | | | | | | | | |■| | |■| | |■|■|■| | | | | | | | |
| 9 | B | |■| | | | | | | | |■| | | | | | |■| | | | | | | | | | | | |■| |■|■| | | |■|■| | | | | | | | |
| 10 | B | | | | | | | | | | |■| | | | | |■| | | | | | | | | | | | | |■|■|■|■| | | | |■| | | | | | | | |
| 11 | B | | | | | | |■| | | | | | | | | | | | |■| | | | | | | | | | |■| |■|■|■| |■| |■| | | | | | | | |
| 12 | B | | | | | | | | | | |■| | | | | | | | |■| | | | | | | | | | |■|■|■|■| |■|■|■|■| | | | | | | | |
| 13 | C | | | | | | | | | | |■| | | | | | | | |■| | | | | | | | | | |■| |■|■| |■| | |■|■| | | | | | | |
| 14 | B | | | | | | | | | | |■| | | | | |■| | | | | | | | | | | | | |■| |■|■|■| |■|■|■|■| | | | | | | |
| 15 | B | | |■| | | | | | | | | | | | | |■| | | | | | | | | | | | | |■| | |■|■| |■|■|■| | | | | |■| | |
| 16 | B | | | | | | |■| | | | | | | | |■| | | | | | | | | | | |■| | |■| |■|■| | |■|■|■| | | | | |■| | |
| 17 | P |■| | | | | | | | | |■| | | | |■| | | | | |■| | | | | | | | |■| | |■|■| | |■|■| | | | | | | | |
| 18 | C | | | | | | | | | | |■| | | | | | |■| | | | | | |■| | | | | |■|■|■|■| | |■| |■| | | | | |■| | |
| 19 | P | |■| | | | | |■| | |■| | | | | | | | |■| | | | | | | | | | |■| | |■| |■|■| |■| | | |■| | | | |
| 20 | C | | | | | | | | | | |■| | | | |■| | | | | |■| | | | | | | | |■| |■|■|■|■| |■|■| | | | | |■| | |
| 21 | C | | | | | | | | | | |■| | | | | |■| | | | | | | | | |■| | | |■| | |■| |■| |■|■| | | | | | | | |
| 22 | P | |■| | | | | | | | | |■| | | | | | | |■| | | | | | | | | |■|■| |■|■| |■|■| |■| | | | | | | | |
| 23 | C | |■| | | | | | | | | |■| | | |■| | | | | | | | | | | | | | |■| |■|■|■| | |■|■| | | | | | | | |
| 24 | C | | | | | | | | | | |■| | | | |■| | | | | |■| | | | | | | | |■| |■|■| | |■|■|■| | | | | | |■| |
| 25 | P | | | | | | | | | | |■| | | | |■| | | | | | | | | | | | | | | |■|■|■| |■|■|■| | | | | | | | |
| 26 | C |■| | | | | | | |■| | | | | | | | | | |■| | | | | | | | | | |■| | |■| | |■|■|■| | | | | | | | |
| 27 | B | |■| | | | | |■| | | | | | | | |■| | | | | | | | | | | | | |■| | |■| | | |■|■| | | | |■| | | |
| 28 | C | | | | | | | | | | | | | | |■|■| | | | | | | | | | | | | | |■| | |■| | | |■|■| | | | | | | |■|
| 29 | C | | | | | | |■| | | | | | | | | | | | | |■| | | | |■| | | | |■| |■|■| | |■|■|■| | | |■| | | | |
| 30 | C | | | | |■| | | | | | | | | |■| |■| | | | | | | |■| | | | | |■| | |■| | | |■|■| | | | | | | | |
| 31 | C | | | | | | |■| | | | | | | | | | | |■| | | |■| | | | | | | |■| |■|■|■| |■|■|■| | | | | | | | |
| 32 | C | | | | | | | | | | | | | | |■| | | | | |■| | | | | | | | | |■| | |■| | |■| |■| | | | | |■| | |
| 33 | C | | | | | |■| | | | | | | | |■| | | | | | | | | | | | | | | |■| | |■| | |■| |■| | | | | | | | |
| 34 | C | | | | | | |■| | | | | | | | |■| | | | | | | | | | | |■| | |■| |■|■| | |■| |■| | | |■| | | | |
| 35 | C | | | | |■| | | | | | | | | |■| | | | | | | | | | | | | | | |■|■| |■|■| | | |■| | | | | | | | |
| 36 | C | | | | | | | | | | | | | | |■| | | | | |■| | | | | | | |■| |■|■| |■|■| |■| |■| | | | | | | | |
| 37 | C | | | | | | | | | | | | | | |■| | | | | | | |■| | | | | | | |■| | |■| | | | |■| | | |■| | | | |
| 38 | C | | | | | | | | | | | | | | |■|■| | | | | | | | | | | | | | |■| | |■| | | | |■| | | | | | |■| |
| 39 | C | | | | | | | | | | | | | | |■|■| | | | | | | | | | | | | | |■|■| |■| | | | |■| | | | | | |■| |
| 40 | C | | | | |■| | | | | | | | | |■| | | | | |■| | | | | | | |■| |■| | |■|■| |■| |■| | | | | | | | |
| 41 | C | | | | | | |■| | | | | | | |■|■| | | | | | | | | | | |■| | |■|■|■|■|■| |■| |■| | | | | | | | |
| 42 | C | | | | |■| | | | | | | | | |■| |■| | | | | | | | | | | | | |■| | |■|■| | | |■| | | | | | | | |
| 43 | C | | | | |■| | | | | | | | | |■|■| | | | | |■| | | | | | | | |■|■|■|■| | | | |■| | | | | | | | |
| 44 | C | | | | | | | | | | | | | | |■| | | | | |■| | | | |■| | | | |■| | |■|■| |■| |■| | | | | | | | |
| 45 | C | | | | | | | | | | | | | | | | | | | | |■| | | | |■| | | | | | | |■|■|■|■| |■| | | | | | | | |
| 46 | C | | | | | | | | | | | | | | | | |■| | | | | | | | | | | | | |■| | |■|■|■|■| |■| | | | | | |■| |
| 47 | C | | | | |■| | | | | |■| | | |■|■| | | | | | | | | | | | | | |■| | |■|■| | | |■| | | |■| | | | |
| 48 | C | | | | | | | | | | | | | | | | |■| | | | | | | | | | | | | |■| | |■|■| |■|■|■| | | | | | | | |

[1] B: beef; P: pork; C: chicken

[2] Conjugation frequencies: Average of three independent experiments

**Figure S5**. Phenotypic and genotypic characteristics of isolates from farm origin (*n*=20)

| Nº | Source[1] | Beta-lactam gene: TEM-1 | TEM-42 | TEM-55 | TEM-116 | TEM-171 | TEML-278 | CTX-M1 | CTX-M2 | CTX-M8 | CTX-M9 | CTX-M14 | CTX-M15 | CTX-M55 | OXA-1 | SHV-12 |
|---|---|---|---|---|---|---|---|---|---|---|---|---|---|---|---|---|
| 1 | S | ■ | | | | | | | | | | | | | | ■ |
| 2 | S | ■ | | | | | | ■ | | | | | | | | |
| 3 | S | | ■ | | | | | | | | | ■ | | | | |
| 4 | S | | ■ | | | | | | | | | ■ | | | | |
| 5 | S | ■ | | | | | | ■ | | | | | | | | |
| 6 | S | | ■ | | | | | ■ | | | | | | | | |
| 7 | S | ■ | | | | | | | | | | ■ | | | | |
| 8 | S | ■ | | | | | | ■ | | | | | | | ■ | ■ |
| 9 | S | | | | | | | | | | | | | | | ■ |
| 10 | S | | | | | | | | | | | ■ | | | | |
| 11 | S | | | | | | | | | | | ■ | | | | |
| 12 | S | | ■ | | | | | | | | | | ■ | | | |
| 13 | S | | | | | | | ■ | | | | | | | | |
| 14 | S | | ■ | | | | | ■ | | | | | | | | ■ |
| 15 | F | | | | | | | ■ | | | | | | | | |
| 16 | F | | | | | | | ■ | | | | | | | | |
| 17 | F | | | | | | | ■ | | | | | | | | |
| 18 | F | | | | | | | ■ | | | | | | | | |
| 19 | F | | | | | | | | | | | ■ | | | | |
| 20 | F | | | | | ■ | | | | | | ■ | | | | |

| Nº | Phylogroup: A | B1 | B2 | C | D | F | MLST: ST10 | ST23 | ST101 | ST168 | ST446 | ST883 |
|---|---|---|---|---|---|---|---|---|---|---|---|---|
| 1 | | | ■ | | | | | | | | | |
| 2 | | | ■ | | | | | | | | | |
| 3 | ■ | | | | | | ■ | | | | | |
| 4 | ■ | | | | | | ■ | | | | | |
| 5 | ■ | | | | | | | ■ | | | | |
| 6 | ■ | | | | | | | ■ | | | | |
| 7 | | | ■ | | | | | | | | | |
| 8 | | | ■ | | | | | | | | | |
| 9 | | | ■ | | | | | | | | | |
| 10 | | | ■ | | | | | | | | | ■ |
| 11 | | | ■ | | | | | | | | | ■ |
| 12 | ■ | | | | | | ■ | | | | | |
| 13 | | | ■ | | | | | | ■ | | | |
| 14 | ■ | | | | | | | | | ■ | | |
| 15 | | ■ | | | | | | | | | | |
| 16 | | ■ | | | | | | | | | ■ | |
| 17 | | ■ | | | | | | | | | ■ | |
| 18 | | ■ | | | | | | | | | ■ | |
| 19 | ■ | | | | | | | ■ | | | | |
| 20 | | | | ■ | | | | | | | | |

| Nº | MGEs: Int1 | IntI2 | *ISECP* 1 | IS26 | IS903 | ISCR1 | Virulence factors: aer | papC | fimA | papG III | cnf1 | hlyA | Conjugation frequency ranges[2]: >1 x $10^{-4}$ and < 1 x $10^{-5}$ | >1 x $10^{-5}$ and < 1 x $10^{-6}$ | >1 x $10^{-6}$ and < 1 x $10^{-7}$ | >1 x 10-7 |
|---|---|---|---|---|---|---|---|---|---|---|---|---|---|---|---|---|
| 1 | | | | ■ | | | ■ | ■ | ■ | | | | | | | |
| 2 | ■ | | ■ | ■ | ■ | | ■ | ■ | ■ | | | | | | | |
| 3 | ■ | | ■ | ■ | ■ | | ■ | ■ | ■ | | | | | | | |
| 4 | ■ | | ■ | ■ | ■ | | ■ | ■ | | | | | | | | |
| 5 | ■ | | ■ | ■ | | | ■ | ■ | ■ | | | | | | | ■ |
| 6 | ■ | | ■ | ■ | ■ | | ■ | ■ | ■ | | | | | ■ | | |
| 7 | ■ | | ■ | ■ | | | ■ | ■ | ■ | | | | ■ | | | |
| 8 | ■ | | ■ | ■ | | | ■ | ■ | ■ | | | | ■ | | | |
| 9 | | | | ■ | | | | ■ | ■ | | | | ■ | | | |
| 10 | ■ | | ■ | ■ | | | | ■ | | | | | | | ■ | |
| 11 | ■ | | ■ | ■ | | | ■ | ■ | | | | | ■ | | | |
| 12 | ■ | | | ■ | ■ | | ■ | ■ | ■ | | | | | ■ | | |
| 13 | ■ | | ■ | ■ | | | | ■ | ■ | | | | | | | |
| 14 | ■ | | ■ | ■ | ■ | | | ■ | ■ | | | | | | | |
| 15 | ■ | | ■ | ■ | | | ■ | ■ | ■ | | | | | | | |
| 16 | ■ | | ■ | ■ | | | ■ | ■ | ■ | | | | | | | |
| 17 | ■ | | ■ | ■ | ■ | | ■ | ■ | ■ | | | | | | | |
| 18 | ■ | | ■ | ■ | | | ■ | ■ | ■ | | | | | | | |
| 19 | ■ | | ■ | ■ | | | | | ■ | | | | | ■ | | |
| 20 | ■ | | | ■ | ■ | | ■ | | ■ | | | | | | | |

[1] S: farm soil; F: feed

[2] Conjugation frequencies: Average of three independent experiments
